# Clinical Application of Artificial Intelligence in Anesthesiology: A Multicenter Retrospective Comparison Between Human Anesthetic Decisions and Algorithmic Recommendations in Non-Cardiac Surgery

**DOI:** 10.3390/jpm16040222

**Published:** 2026-04-17

**Authors:** Gilberto Duarte-Medrano, Natalia Nuño-Lámbarri, Octavio Gonzalez-Chon, Rebeca Garazi Elguezabal Rodelo, Carmelo Calvagna, Daniele Paternò, Luigi La Via, Massimiliano Sorbello

**Affiliations:** 1 Anesthesiology Department of Hospital Medica Sur, Mexico City 14050, Mexico; ogchon@medicasur.org.mx (O.G.-C.); rbk.elguezabal27@gmail.com (R.G.E.R.); 2 Translational Research Unit, Medica Sur Clinic & Foundation, Mexico City 14050, Mexico; 3 Department of Surgery, Faculty of Medicine, The National Autonomous University of Mexico (UNAM), Mexico City 04510, Mexico; 4 Department of Anesthesia and Intensive Care, University Hospital Policlinico “G. Rodolico–San Marco”, 95121 Catania, Italy; gicalvagna@tiscali.it (C.C.); luigi.lavia@unict.it (L.L.V.); 5 Department of Anesthesia and Intensive Care, Hospital “Giovanni Paolo II”, ASP Ragusa, 97100 Ragusa, Italy; paternomd@icloud.com; 6 Department of General Surgery and Medical Surgical Specialties, University of Catania, 95131 Catania, Italy; 7 Faculty of Medicine and Surgery, University of Enna “Kore”, 94100 Enna, Italy; massimiliano.sorbello@unikore.it

**Keywords:** anesthesia, IA, ChatGPT, hybrid model

## Abstract

**Background**: Artificial intelligence (AI) is progressively entering perioperative medicine; however, its role in preoperative anesthetic decision-making remains insufficiently characterized. We evaluated the concordance between anesthesiologist-selected anesthetic techniques and algorithm-generated recommendations in a cohort of adult patients undergoing non-cardiac surgery. **Methods**: This retrospective observational study included adult patients (≥18 years) undergoing elective non-cardiac surgery between January 2024 and January 2025 at two international centers (Mexico and Italy). Clinical, demographic, and surgical variables were extracted from electronic medical records. For each case, a structured anonymized vignette was submitted to ChatGPT (version 5.0, medical configuration) to obtain an independent recommendation regarding anesthetic technique. Concordance between AI-generated and clinician-selected techniques was assessed using agreement analysis and stratified by country and surgical specialty. **Results**: A total of 1965 patients were analyzed. Overall concordance between ChatGPT recommendations and anesthesiologist-selected techniques was 84.6%. Agreement remained stable across centers (Mexico 84.3%; Italy 88.7%). Disagreement rates varied by surgical specialty, with the highest values observed in vascular and proctologic surgery (28.6%), followed by urology (21.1%) and thoracic surgery (18.8%). Orthopedic procedures—particularly shoulder arthroscopy—accounted for a relevant proportion of divergences, where AI frequently favored regional techniques over general anesthesia. No specialty demonstrated discordance exceeding 30%. **Conclusions**: AI-generated anesthetic recommendations demonstrated substantial concordance with expert clinical decision-making across heterogeneous surgical settings. These findings support the potential integration of AI within a hybrid decision-making framework, complementing—rather than replacing—anesthesiologist expertise in contemporary perioperative care.

## 1. Introduction

Contemporary anesthesiology faces increasingly complex challenges driven by the growing sophistication of surgical procedures, the heterogeneity of patient populations, and the need for accurate clinical decision-making in highly dynamic perioperative environments. Within this context, artificial intelligence (AI) has emerged as a potential transformative tool, offering the ability to support anesthesiologists across multiple phases of perioperative care [1,2,3,4,5]. Nevertheless, the effective integration of AI into anesthetic practice requires a thorough understanding of its capabilities, limitations, and real-world impact on clinical decision-making. Although the application of AI in anesthesiology remains at an early stage, accumulating evidence suggests promising results in several key domains including prediction of intraoperative adverse events and anesthetic depth monitoring [6,7,8,9,10,11,12]. Interestingly beyond intraoperative management, recent literature highlights the potential role of AI in preoperative anesthetic decision-making [13]. In this setting, generative AI models—such as ChatGPT, Gemini, and PEACH—have been explored as decision-support tools capable of suggesting anesthetic techniques tailored to individual patient characteristics and surgical requirements accurately [13,14,15]. These developments open new perspectives on personalized anesthetic planning, while simultaneously raising important questions regarding reliability, transparency, and clinical accountability. Importantly, the implementation of AI-based systems should be framed within a hybrid decision-making model [16]. Such an approach does not aim to replace human clinical judgment, but rather to augment it through the computational analysis of multiple variables in real time. The anesthesiologist remains the central figure in perioperative management and will benefit from tools capable of providing evidence-informed anesthetic recommendations while preserving professional autonomy and responsibility. In this context, structured education and training in AI-based technologies are essential to ensure their safe, ethical, and effective integration into routine clinical practice.

In recent years, the field of anesthesiology has experienced a rapid expansion in the scope and sophistication of artificial intelligence applications, largely driven by advances in machine learning methodologies and the increasing availability of high-resolution perioperative data. Modern AI systems have evolved beyond static predictive models to incorporate adaptive algorithms capable of integrating multimodal inputs—including continuous physiological waveforms, electronic health records, and intraoperative monitoring signals—in real time. This capability has facilitated the development of highly granular predictive tools for perioperative risk stratification, enabling early detection of adverse events such as intraoperative hypotension, hypoxemia, and hemodynamic instability, and thereby supporting a shift toward anticipatory and precision-guided anesthetic management.

Concurrently, there is a growing trend toward the incorporation of AI into closed-loop and semi-autonomous anesthetic delivery systems. These systems integrate predictive analytics with automated pharmacologic titration—particularly of agents such as propofol and remifentanil—guided by processed electroencephalographic indices and hemodynamic parameters. Preliminary evidence suggests that such platforms may enhance the precision of anesthetic depth control, reduce inter-provider variability, and optimize recovery profiles. However, their implementation also necessitates careful consideration of safety frameworks, including system validation, cybersecurity, and the maintenance of continuous clinician oversight to mitigate potential risks associated with algorithmic limitations or unexpected clinical conditions.

Furthermore, the emergence of large language models and generative AI has extended the role of artificial intelligence from monitoring and prediction to higher-order cognitive support in clinical reasoning. These models demonstrate the capacity to synthesize complex clinical datasets and generate context-specific anesthetic strategies, aligning with the broader paradigm of personalized medicine. Importantly, this evolution is accompanied by increasing emphasis on explainable artificial intelligence, aimed at improving transparency, interpretability, and clinician trust in algorithmic outputs. Collectively, these trends underscore a paradigm shift toward a synergistic model in which human expertise is augmented by AI-driven decision support, highlighting the need for robust validation, regulatory oversight, and standardized training to ensure safe and effective integration into anesthetic practice.

The aim of the present study was to retrospectively evaluate the degree of concordance between anesthetic technique decisions made by anesthesiologists and the recommendations generated by different artificial intelligence applications in adult patients undergoing non-cardiac surgery in Mexico and Italy.

## 2. Materials and Methods

A retrospective observational study was conducted based on the systematic review of electronic medical records. All included patients were adults (≥18 years) scheduled for elective non-cardiac surgery in the operating rooms of Hospital Médica Sur and the University Hospital Policlinico G. Rodolico–San Marco over the study period from January 2024 to January 2025.

### 2.1. Study Design and Setting

The study was designed to evaluate the concordance between anesthetic techniques selected by attending anesthesiologists and those recommended by an artificial intelligence system under standardized conditions. The AI system was applied retrospectively and had no influence on clinical decision-making.

### 2.2. Patient Selection

Patient selection followed predefined inclusion and exclusion criteria to ensure methodological consistency and reproducibility.

Inclusion criteria:

1. Adult patients (≥18 years of age)
2. Undergoing elective non-cardiac surgery during the study period
3. Availability of complete perioperative data required to reconstruct a standardized clinical vignette, including demographic, clinical, and surgical variables

Exclusion criteria:

1. Patients <18 years of age
2. Emergency surgical procedures
3. Incomplete or missing data in any of the key variables required for analysis
4. Cases in which clinical information was insufficient to generate a consistent AI-based evaluation

The final study cohort therefore represents a filtered dataset based on data completeness and suitability for structured AI input, rather than the total surgical volume of each institution. This approach explains the lower number of eligible cases from the Italian center, where data standardization and completeness were more limited.

### 2.3. Data Collection and Variable Definition

A structured database was developed from electronic medical records. The following variables were systematically extracted and coded:

• Demographic data: age, sex
• Anthropometric variables: weight, height, body mass index (BMI)
• Clinical comorbidities: including but not limited to arterial hypertension, diabetes mellitus, chronic obstructive pulmonary disease, and cardiovascular disease
• Substance use history: tobacco, alcohol, and illicit drug use
• Surgical information: surgical specialty responsible for the procedure
• Anesthetic technique performed: as documented in the anesthetic record

Due to variability in documentation across centers, surgical procedures were categorized by specialty rather than by specific procedure type or anatomical location, which was not consistently available in a structured format.

### 2.4. Classification of Anesthetic Techniques

Anesthetic strategies were classified into three predefined categories to allow standardized comparison:

1. General anesthesia: including total intravenous anesthesia (TIVA) and balanced inhalational anesthesia
2. Regional anesthesia: including neuraxial techniques (spinal, epidural, combined spinal-epidural) and peripheral nerve blocks (single-shot or continuous)
3. Combined anesthesia: any combination of general anesthesia with regional techniques

This classification reflects strategy-level decision-making, rather than procedural-level technical granularity.

### 2.5. AI-Based Anesthetic Assessment

Following data extraction and ethical approval, each patient case was converted into a standardized, anonymized clinical vignette. These vignettes were constructed using only variables available in the database, ensuring consistency across all cases.

The dataset from both centers was unified prior to AI analysis. Each vignette was then independently analyzed using a customized configuration of ChatGPT (version 5.0), referred to as “Anesthetic Assessment.” The AI system was prompted using a fixed, standardized instruction applied uniformly to all cases, as follows:

“Based on the following clinical scenario, determine the most appropriate anesthetic technique (general, regional, or combined anesthesia), considering patient characteristics, comorbidities, and type of surgery, in accordance with current anesthesiology guidelines.” This approach represents a structured prompting strategy, not a deterministic or rule-based algorithm. The model integrates the provided variables to generate a recommendation simulating clinical reasoning based on its training in medical literature and guidelines.

AI outputs were restricted to:

• A single recommended anesthetic strategy (general, regional, or combined)
• A brief supporting rationale (used qualitatively but not included in quantitative analysis)

Importantly, the AI system had no access to the actual anesthetic technique performed, ensuring independence of assessment.

### 2.6. Definition of Concordance

Concordance was defined as agreement between the anesthetic strategy selected by the attending anesthesiologist and the recommendation generated by the AI system at the categorical (strategy) level.

• Concordant cases: same category (general vs. regional vs. combined)
• Discordant cases: different categories between AI and clinician

No weighting was applied, and agreement was assessed as a binary outcome.

### 2.7. Methodological Considerations and Limitations

Several methodological considerations inherent to the retrospective design should be acknowledged:

• Patient preference was not systematically documented and therefore not included in the AI input
• Institutional factors, such as resource availability or clinician expertise, were not captured
• Anatomical feasibility of regional techniques could not be assessed due to lack of procedural-level detail
• The analysis was conducted at a strategy level, not accounting for specific anesthetic techniques (e.g., type of nerve block)

These factors may influence both clinical decision-making and observed concordance.

## 3. Results

### Baseline Characteristics

A total of 1965 patients were included in the analysis, the majority of whom were treated in Mexico (92.4%), with the remaining 7.6% managed in Italy. Overall, demographic, anthropometric, biochemical, hematological, and coagulation profiles were well balanced between the two cohorts, indicating a broadly comparable baseline clinical phenotype across countries. A comprehensive summary of baseline characteristics is provided in Table 1.

Regarding surgical specialty, orthopedic and general surgery represented the largest proportion of procedures, collectively accounting for 57.8% of the cohort (32.4% and 25.4%, respectively). Cardiovascular comorbidities were the most prevalent chronic conditions, with arterial hypertension observed in 28.7% of patients and a history of arrhythmias or ischemic heart disease in 7.8%. Diabetes mellitus was present in 12.7% of cases. Pulmonary comorbidities—including obstructive sleep apnea, chronic obstructive pulmonary disease, and asthma—were documented in 9.5% of patients.

Agreement between ChatGPT and anesthesiologists by country

**Figure 1:**
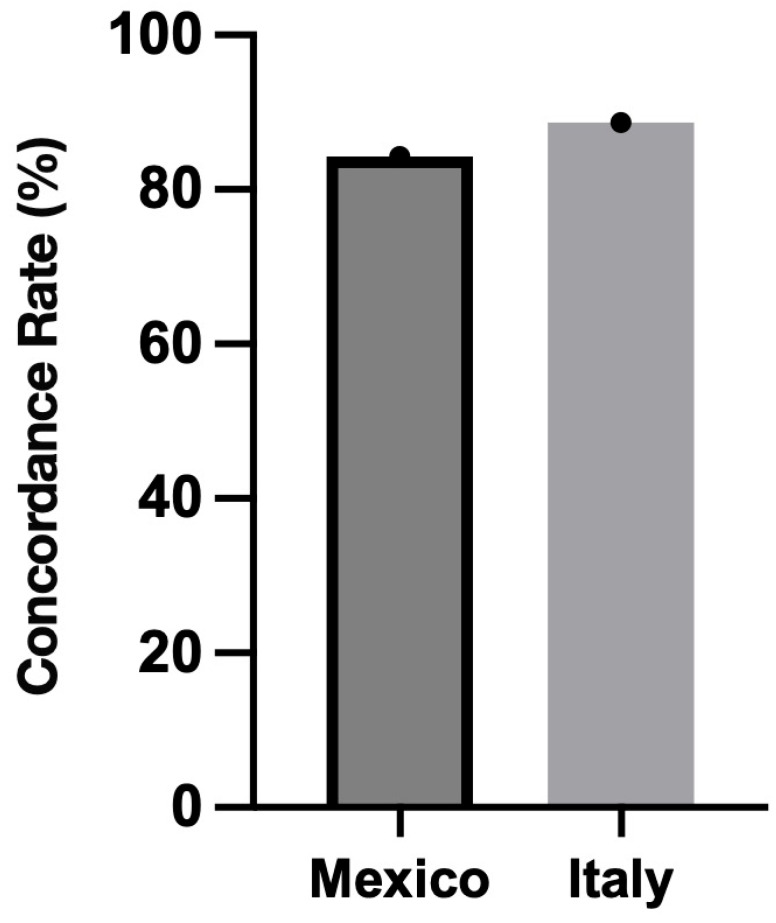
Concordance analysis of ChatGPT-generated anesthetic recommendations versus human clinical decisions across the two international centers.

Across the entire cohort, the concordance between ChatGPT-generated recommendations and anesthesiologist-selected anesthetic techniques reached 84.6%. When stratified by country, agreement remained consistently high, at 84.3% in Mexico and 88.7% in Italy (Figure 1). These findings suggest a robust alignment between AI-assisted recommendations and expert clinical decision-making across distinct healthcare systems.

Agreement between ChatGPT and anesthesiologists by surgical specialty

The degree of agreement between ChatGPT and anesthesiologists varied according to surgical specialty. The highest rates of disagreement were observed in proctologic and vascular surgery (both 28.6%), followed by urology (21.1%), thoracic surgery (18.8%), and orthopedic surgery (17.9%). Intermediate disagreement rates were noted in head and neck surgery (15.3%), oncologic surgery (13.7%), hepatopancreatobiliary surgery (12.9%), plastic surgery (12.1%), and neurosurgery (9.4%).

Lower disagreement rates were identified in general surgery (7.6%), while gynecologic surgery demonstrated moderate variability (25.0%). Notably, complete agreement between ChatGPT and anesthesiologists was observed, in endocrine surgery, marking a new high. All discrepancies are shown in Table 2.

**Table 2:** Disagreement Rates Between ChatGPT and Anesthesiologists by Surgical Specialty.

Surgical Specialty	*N* (Patients)	Disagreement Rate (%)
Orthopedic surgery	637	17.9
General surgery	500	7.6
Oncologic surgery	131	13.7
Hepatopancreatobiliary surgery (HPB)	31	12.9
Urology	209	21.1
Thoracic surgery	48	18.8
Head and neck surgery	85	15.3
Proctologic surgery	133	28.6
Vascular surgery	35	28.6
Neurosurgery	96	9.4
Plastic surgery	33	12.1
Gynecologic surgery	8	25.0
Endocrine surgery	22	0.0 (100% agreement)

## 4. Discussion

The present multicenter retrospective analysis demonstrates a high degree of concordance between anesthesiologist-selected anesthetic techniques and ChatGPT-generated recommendations, exceeding 80% across two international centers. This finding is particularly relevant, as it reflects not only the robustness of the model’s internal reasoning architecture but also its alignment with contemporary international anesthesiology guidelines that likely informed its training framework. Importantly, the observed concordance rate in our large-scale cohort (*n* = 1965) surpasses that reported by Çelik et al. [17], who described substantially lower agreement in a smaller single-center population. This difference may be attributable to the broader clinical heterogeneity, greater surgical diversity, and updated AI configuration employed in our study, thereby offering a more externally valid evaluation of AI-supported anesthetic decision-making.

Nevertheless, specific areas of divergence deserve careful interpretation. Orthopedic surgery—particularly shoulder arthroscopy—represented one of the principal sources of discordance in our cohort. In this subgroup, the AI model frequently recommended ultrasound-guided peripheral nerve blocks (supraclavicular or interscalene approaches), whereas anesthesiologists often opted for general anesthesia. From a purely evidence-based perspective, the AI’s preference may reflect adherence to enhanced recovery principles and contemporary regional anesthesia guidelines favoring opioid-sparing strategies. However, real-world decision-making incorporates additional variables that may not be fully captured in structured datasets: institutional workflow constraints, operating room turnover considerations, variability in regional anesthesia expertise, patient refusal of awake techniques, or legal and logistical factors. These nuances illustrate a fundamental distinction between algorithmic optimization and pragmatic clinical practice.

A similar pattern emerged in urologic procedures, where moderate discrepancies were observed. Here again, patient preference, surgical team expectations, positioning requirements, and anticipated procedure duration may influence the final anesthetic choice. Importantly, despite these divergences, no surgical specialty demonstrated a discordance rate exceeding 30%, with the highest values observed in vascular and proctologic surgery (28.6%). This ceiling effect suggests that the AI model maintained clinically coherent recommendations across a wide spectrum of surgical subspecialties, reinforcing its structural reliability as a decision-support tool.

Notably, in contrast to previously published experiences, our model appeared capable of effectively integrating patient-specific variables—including laboratory parameters and comorbidities—into its recommendations. This may reflect advances in model training, contextual reasoning capabilities, or improved prompt structuring. The ability to process complex multimodal inputs and synthesize them into a coherent anesthetic strategy underscores the potential of large language models as cognitive augmentation tools rather than mere information retrieval systems.

Among the principal strengths of our investigation is its multicenter international design, encompassing a substantially larger population than earlier studies (1965 vs. 72 patients) [17]. The inclusion of diverse surgical subspecialties enhances generalizability and reflects the complexity of contemporary anesthetic practice. Furthermore, the use of an updated AI configuration allows a more realistic assessment of current-generation generative models in clinical decision simulation.

However, several limitations warrant acknowledgment. First, the retrospective design inherently limits the ability to capture contextual factors influencing anesthetic decisions, such as dynamic intraoperative considerations, patient anxiety, or informal interdisciplinary discussions. Second, the analysis focused on a single AI system; comparative evaluation with additional models could further delineate performance variability. Third, the study assessed concordance rather than clinical outcomes; future investigations should explore whether AI-aligned decisions translate into measurable improvements in perioperative endpoints, such as hemodynamic stability, analgesic consumption, or recovery profiles.

From a broader perspective, our findings reinforce that the integration of artificial intelligence into anesthesiology represents not merely a technological advancement, but a critical step toward the realization of precision and personalized perioperative medicine. By enabling the simultaneous analysis of multiple patient-specific variables—such as comorbidities, physiological status, and surgical context—AI systems have the potential to support individualized anesthetic strategies tailored to each patient’s unique risk profile.

However, the successful translation of these tools into clinical practice requires careful alignment with updated international guidelines, perioperative optimization protocols, and continuous model recalibration. Importantly, AI-driven recommendations should be integrated within a patient-centered framework, where clinical judgment, patient preferences, and contextual factors remain central to decision-making.

Future developments should focus on dynamically training AI systems to incorporate evolving evidence, including advancements in regional anesthesia, anticoagulation management, and enhanced recovery pathways, while also integrating patient-reported outcomes and shared decision-making processes. In this context, artificial intelligence may serve as a key enabler of a hybrid, personalized anesthetic model, enhancing both the precision and consistency of perioperative care while preserving the essential role of the anesthesiologist.

## 5. Conclusions

The integration of artificial intelligence into anesthesiology is not a hypothetical evolution but an emerging reality. However, its meaningful incorporation into clinical practice requires careful alignment with contemporary international guidelines, perioperative optimization protocols, and evidence-based advancements in anesthetic techniques. When integrated within a structured framework that preserves professional judgment, ethical responsibility, and individualized patient assessment, AI has the potential to contribute to a hybrid decision-making model—one in which computational insight and human expertise coexist synergistically.

## Figures and Tables

**Table 1:** Characteristics of the study population.

Variable	Mexico (*n* = 1815)	Italy (*n* = 150)
Age (years)	54.3 ± 17.2	58.7 ± 17.6
Hemoglobin (g/dL)	14.5 ± 2.1	14.3 ± 2.3
Hematocrit (%)	42.3 ± 6.0	41.7 ± 6.0
Platelets (×10^3^/µL)	262.5 ± 89.4	257.1 ± 97.2
Leukocytes (/µL)	7915 ± 6026	7623 ± 3655
Glucose (mg/dL)	102.8 ± 26.3	106.3 ± 25.3
Blood urea nitrogen (mg/dL)	17.1 ± 7.1	17.4 ± 8.2
Creatinine (mg/dL)	0.9 ± 0.3	0.9 ± 0.4
Total protein (g/dL)	10.8 ± 2.1	10.7 ± 1.8
INR	1.0 ± 0.1	1.0 ± 0.1
Activated partial thromboplastin time (s)	30.8 ± 4.3	30.9 ± 4.1
Weight (kg)	74.4 ± 16.7	74.2 ± 14.8
Height (m)	1.7 ± 0.1	1.7 ± 0.1
Body mass index (kg/m^2^)	26.6 ± 5.1	26.8 ± 4.6

## Data Availability

The data used in this study were derived from retrospective databases belonging to Hospital Médica Sur and the University Hospital Policlinico G. Rodolico. Due to institutional policies and data protection regulations, these datasets contain identifiable patient information and cannot be publicly shared. Access to the data may be considered upon reasonable request, subject to approval by the respective institutional review boards and compliance with applicable confidentiality and privacy regulations.

## Abbreviations

The following abbreviations are used in this manuscript:

AI	Artificial Intelligence
ASA	American Society of Anesthesiologists (Physical Status Classification)
BMI	Body Mass Index
COPD	Chronic Obstructive Pulmonary Disease
EEG	Electroencephalography
HPB	Hepatopancreatobiliary
INR	International Normalized Ratio
SPSS	Statistical Package for the Social Sciences
